# Editorial: Visual perception and mental imagery in aging, health and disease

**DOI:** 10.3389/fpsyg.2026.1812355

**Published:** 2026-04-14

**Authors:** Anna Maria Berardi, Renaud F. Cohen, Ofelia Tatu

**Affiliations:** 1 LCOMS, Département de Psychologie, UFR SHS-Metz, Université de Lorraine, Metz, France; 2 Centre Hospitalier Regional Universitaire de Nancy Hopitaux de Brabois, Vandœuvre-lès-Nancy, France; 3 Crisis and Trauma Resource Institute, Winnipeg, MB, Canada

**Keywords:** dorsal, mental imagery, pathway, perception, ventral, visual

Most previous studies have focused on the role of the dorsal and ventral pathways in visual perception, but fewer have examined their role in mental imagery. However, visual perception and mental imagery share much of the same brain circuitry. In visual perception, after visual input, the visual characteristics of objects and their locations in space, are analyzed in the ventral and dorsal visual pathways, respectively. In mental imagery, the visual characteristics of objects and their locations have to be reconstructed and visualized as if the objects were physically present. Through this Research Topic, we sought to further our understanding of (1) the brain circuits involved in visual perception and mental imagery and (2) the roles of the dorsal and ventral visual pathways in visual perception and mental imagery. It was the aim of this Research Topic to invite investigators to submit articles relevant to dorsal and ventral visual processing for both visual perception and mental imagery in humans, as well as to further our understanding of the relationships between low-level and high-level visual processes.

The articles submitted attest to the wide variety of contributions to the topic, the variety of techniques that have been used to further our understanding of visual perceptual and mental imagery functions, and the richness of the advances in the field. Eight manuscripts have been published in the Research Topic “*Visual perception and mental imagery in aging, health and disease*,” attesting to the interest for this topic and the variety of the scientific contributions.

Szaszko et al. used brain evoked potentials to show that brain rhythms in the alpha range (10 Hz) help participants ignore distracting and irrelevant color cues in a visual conjunction search task. Results showed both electrophysiological and behavioral evidence of suppression. Indeed, an event-related brain evoked potential typically associated with suppression confirmed suppression during rhythmic but not non-rhythmic stimulations. The study demonstrates clear evidence of suppression after rhythmic flickering, although behaviorally, the participants did not show improved performance.

Wei et al. used psychophysical techniques to assess low-level visual function in amblyopes compared to controls. The balanced Contrast Sensitivity Function (CSF) algorithm was used to stimulate the ON/OFF pathways unselectively, and CSFs for increments and decrements were used to selectively stimulate the ON/OFF visual pathways. Results show that for the balanced CSF, there were significant

interocular differences in both amblyopes and controls, although they were more pronounced for amblyopes. Moreover, the results in the CSF increment condition were significantly lower than those in the decrement condition in the amblyopic eye. These differences in increment and decrement CSFs were not observed in the fellow eye of amblyopes or in controls, suggesting subtle differences in contrast sensitivity for the amblyopic eye when exposed to stimulation of the ON/OFF pathways.

Marczak-Czajka et al. used Convolutional Neural Networks (CNN) to generate abstract images and study how they affect human emotions. Results suggested that some images, due to their hue or feature configuration, increased arousal, while leaving valence unmodified. The results suggest that generating visual images with neural networks could be used as a way to influence emotion and help supporting mental health in a non-invasive manner.

Demas demonstrated a first case of Alice In Wonderland Syndrome (AIWS) in a patient with Dementia with Lewy Bodies (DLB) who presented with a brief episode of misperception, reporting that his bed had “shrunk.” These results suggest a dysfunction of high-level visual processes in the right extrastriate cortex, a region responsible for the storage of canonical object sizes. An impairment in this region appears to lead to an agnosia for object sizes. The results also suggest that AIWS should be considered among the visual impairments of DLB.

Goto et al. examined how eye movements, heart rate and mood, contribute to the reduction of stress when participants look at a well-structured and well-maintained japanese garden and a control garden without such properties. In the Murin-An garden participants' eyes moved faster and explored a wider area than in the control garden. They also showed lowered heart rate and improved mood compared to the control garden. However, stress levels were not linked to looking at specific objects. The results suggest that stress reduction is not associated to specific elements of the gardens but to their overall design. The Murin-An garden encourages frequent and rapid eye-movements, which appears to be the main reason for a reduction in stress, similar to the rapid eye-movements generated in Eye Movement Desensitization and Reprocessing therapy.

Miki et al. reviewed EEG studies of the brain processes involved in face perception. Most previous research on face perception has been carried out in Western countries and show that face perception in children changes as they grow. The authors focus on face perception in Japanese children and compare these results to those of western children. Japanese children show adult-like EEG responses at age 13, an earlier age than western children, although the recognition of facial emotions in Japanese children continues to improve after age 14. Moreover, expertise appears to improve attention to facial emotions and influence early stages of face processing. These results suggest that face perception may develop at different speeds depending on the specific functions assessed and may vary according to cultural background and personal expertise.

Berardi presents the Imagery Processing Battery (IPB), a new test battery of 15-tasks relying predominantly on the dorsal or ventral visual pathways, for assessing different and comparable aspects of visual perception and mental imagery abilities. This study shows that in young participants, the dorsal and ventral visual pathways are well-segregated at the behavioral level for both perceptual and mental imagery conditions.

Berardi further shows, by using the 15-tasks IPB battery, described in detail in the previous publication, that the dorsal and ventral visual pathways are not as well-segregated at the behavioral level for visual perceptual and mental imagery conditions equated for difficulty, in old as compared to young participants.

The results presented in this Research Topic are important. Future research should be made to further improve our understanding of the dorsal and ventral visual pathways and their interactions in healthy participants and in participants with brain pathologies, using a wide variety of techniques, such as those used by the authors included in this Research Topic.

